# Molecular Analysis and Bioinformatics Assessment of Full-Length L1 Gene of Bovine Papillomavirus Type-1 as a Potential DNA Vaccine Study

**DOI:** 10.1155/vmi/6785087

**Published:** 2025-04-10

**Authors:** Hassan Hadi Abdul-Zahra, Yahia I. Khudhair, Husam Raheem Al-Hraishawi

**Affiliations:** ^1^Hillah Veterinary Hospital, Ministry of Agriculture, Hillah, Iraq; ^2^Department of Internal and Preventive Medicine, College of Veterinary Medicine, University of Al-Qadisiyah, Al-Diwaniya, Al-Qadisiyah, Iraq; ^3^Department of Physiology, College of Medicine, University of Misan, Amarah, Maysan, Iraq

**Keywords:** *Bovine papilloma*, epitopes, in silica analysis, Iraq, phylogeny

## Abstract

**Background:** Papillomaviruses (PVs) infect animals and humans and are linked to 27%–30% of cancers. The L1 protein is a cornerstone in bovine PVs (BPVs), being the main components of the viral capsid and playing pivotal roles in infectivity and antigenicity.

**Objective:** The current study aims to characterize the genetic variation in the L1 gene of the BPV, explore in silico the protein structure, predict epitopes, and evaluate the impact of mutation on the epitope conservancy.

**Methods:** Fifty tumor samples were collected from cattle with papilloma lesions from Babylon, Wasit, and Al-Qadisiyah provinces, Iraq. Samples were submitted to PCR to amplify the complete L1 gene. Phylogeny was performed to assess the L1 gene. Various bioinformatics tools were utilized to analyze physicochemical properties, secondary structure of the deduced protein, and predict immunodominant epitopes for B and T cells.

**Results:** BPV DNA was detected in 42 (84%) samples. Sequence analysis of 10 samples revealed that BPV-1 was the predominant type circulating in study regions. Phylogeny demonstrated that analyzed strains were aligned with a distance value of 1%–15% to strains of delta PVs. Amino acid characterization indicated two amino acid mutations compared with reference strain (X02346.1) including SER31/ASN and Ala 55/ASP. The 3D model revealed L1 that formed from hexameric subunits, each subunit with six loops. ALA 55/ASP substitutions are located in the Loop1. The predicted B- and T-cell epitopes showed that L1 protein has highly potent epitopes and can be a promising target for nucleic acid vaccine design to elicit an anti-BPV humeral and cellular immune response.

**Conclusions:** The current investigation has provided crucial insights into BPV-1 type and diversity in the middle provinces of Iraq. These predominant strains have been identified and registered at NCBI for the first time. The amino acid mutations in the L1 protein have been highlighted. The conserved T- and B-cell epitopes that can detect BPV-1 type have been stablished. Finally, this project is the initial phase of creating a DNA-based vaccination for preventative and treatment purposes against BPV-related illnesses.

## 1. Introduction

Papillomaviruses (PVs) are a diverse group of viruses that infect a wide variety of animal species, including humans, and have been associated with 27%–30% of different cancer types [1, 2]. Interestingly, PVs are highly species-specific. These viruses can initially infect keratinized epithelia and mucosal cells, leading to benign and hyperplasic lesions in most creatures [3–5]. Furthermore, bovine PVs (BPVs) have been implicated not only as oncogenic but also as common health problems in cattle farms [6]. These infections can lead to massive economic losses in the livestock industry due to leather depreciation and mortality. Surprisingly, cattle, the most common species in the animal kingdom, are highly volatile to BPV infection [7]. Numerous investigators have confirmed that the BPV can infect both domestic and wild animals. BPV also causes equine sarcoid in horses and donkeys [2].

Despite having a relatively small genome, BPVs show a high degree of genetic variation, with up to 29 genotypes (BPV-1–BPV-29) that result from the Gene L1 variety [8, 9]. Five genera have been identified thus far. The BPVs genera include delta PV (BPV-1, BPV-2, BPV-13, and BPV-14), xi PV (BPV-3, BPV-4, BPV-6, BPV-9, BPV-10, BPV-11, BPV-12, BPV-15, BPV-17, BPV-20, BPV-23, BPV-24, BPV-26, BPV-28, and BPV-29), epsilon PV (BPV-5, BPV-8, and BPV-25), dyoxi PV (BPV-16, BPV-18, and BPV-22), and dyokappa PV (BPV-19, BPV-21, and BPV-27) [10–13]. The majority of known BPVs are nonenvelop, tiny viruses with a circular double-stranded DNA (dsDNA) genome that ranges in size from 5.8 to 8.6 kb [14]. The BPV genome is composed of three distinct regions. The first region expresses seven nonstructural proteins involved in viral genome replication (early proteins, acronym: E1–E7). The second region encodes the two capsid proteins (later proteins, acronym: L1 and L2) that form a protein capsid that encapsulates the viral genome [15]. One of the main capsid proteins, L1 is highly immunogenic and expressed at constant levels, making it an ideal candidate for the development of a BPV vaccine [16]. In Iraq, the BPV-1 genotype has been identified in nearly 86% of cutaneous and mucosal cancers [17]. Additionally, a significant level of antibodies against PV proteins in various animal species suggests an early stage infection [18]. These data demonstrate the urgent need for a specific vaccine to eradicate BPVs in Iraq.

Vaccination against human PV (HPV) has become a public health major priority, and these vaccines can help prevent HPV infection and may result to treat precancerous lesions of cervical and genital warts, especially in adolescents [19]. Furthermore, in the field of human medicine, HPV vaccinations—both quadrivalent and bivalent—have been implemented into numerous national and international initiatives in many countries worldwide [20, 21]. Recently, recombinant papillomavirus-like particles (VLPs) have been commercially produced via expression based on the HPV-L1 gene through yeast or insect cultures [22]. In addition, VLPs have considerable avenues and potentialities to induce specific immune responses [23]. Several subunit vaccines are under development, including edible, mucoadhesive polymer-based, chimeric, multivalent, and biodegradable vaccines [24]. More importantly, DNA vaccines are thought to be a stable, affordable, safe, and effective approach to preventing BPV infection and can also be used as a good model for research on human recombinant vaccines [25, 26].

When developing a novel vaccination based on genetically engineered viruses encodes full-length viral antigens, the effectiveness of the vaccine has been assessed using both humoral and cellular immune responses [27]. Breakthroughs in immunotherapy, nucleic acid recombinant technology, and molecular biology have provided excellent potential for the design and production of efficient therapeutic and preventative vaccines [28]. The challenges of traditional vaccines production arising from time consumed and cost, that necessitate the development of innovative vaccine. These types of vaccines can have produced from subunit protein, mRNA, or plasmid DNA [29]. BPVs express several gene products, and their contribution in host protection is undefined [25]. Some of these gene products are immune-modifier agents, whose function and impact in vaccinated animals are unknown. It will be necessary to improve molecular studies and bioinformatics characterization of the target major viral compartment protein to generate a better-defined DNA vaccine that will stimulate producing protective antigens. Thus, the current study's objectives were to pre–in silico analyze the L1-BPV-1 protein in order to investigate its 3D structure and predict its immunological characteristics, which can aid in the in vitro and in vivo evaluation of the L1 protein's immunotherapy properties.

## 2. Materials and Methods

### 2.1. Clinical Presentation and Sample Collection

An investigative study was conducted to collect 50 skin samples from 50 cow—suspected of having papillomatosis (warts)—of different ages and sexes in various regions of Babylon, Wasit, and Diwaniyah/Iraq, between September and November/2023. Tumor morphology was used to identify papilloma-like warts during sampling. The project was approved by the ethical committee of council of college of veterinary medicine, University of Al-Qadisiyah registration under Proposal Ref. 1890 on 28/8/2023. Warts samples were surgically removed after obtaining the owner's consensus. The wart samples were put in a sterilized suitable container, and labeled and then transported in a cooled manner to the laboratory. Here, the hair was removed and the samples were washed carefully with phosphate-buffered saline (PBS) and stored at −80°C until it was used.

### 2.2. DNA Extraction and Molecular Detection

Tumor samples were ground with liquid nitrogen. Two grams of ground tumor tissue was used for DNA extraction. DNA extraction was performed using the gSYNC viral DNA/RNA Extraction Kit (Geneaid, Taiwan) according to the manufacturer's instructions. The extracted DNA was quantified and stored at −20°C until further use. PCR reaction was conducted with specific primers designed for L1 gene: the forward primer, 5′-catgcGGATCCctaattttttttgcagaccatggcgttgtggc-3′; and L1 reverse primer, 5′-aggtgcTCGAGcaggttgacttaccttctg-3′. The underlined letters indicate *Bam*HI and *Xho*I restriction sites, and the forward primer was optimized to adding a Kozak consensus sequence (double underlined sequence) (ACC) by using APE-A plasmid editor software (APE-A plasmid editor v3.1.3) at forward and reverse primers, respectively. The primers were designed based on complete genome sequences of BPV deposited in the GenBank (Accession Nos. X02346, NC_001522, AB626705, and JX678969).

The PCR reactions were carried out using a 2X PCR MasterMix kit (ABM, Canada) in 50 μL reaction volume, consisting of 25 μL 2X PCR MasterMix, 1.5 μL of each primer (10 pmol), 5 μL of DNA template, and 17 μL of nuclease-free water. The reaction condition included the initial denaturation step of 95°C/5 min, followed by 35 cycles of 95°C/30 s of denaturation, 55°C/30 s of annealing, 72°C/90 s of extension, and finally 72°C/7 min of final extension. The PCR products were electrophoresed on a 1% ethidium bromide-stained agarose gel and then visualized under UV light.

### 2.3. Preparation and Cloning of L1 Gene

**Figure 1:**
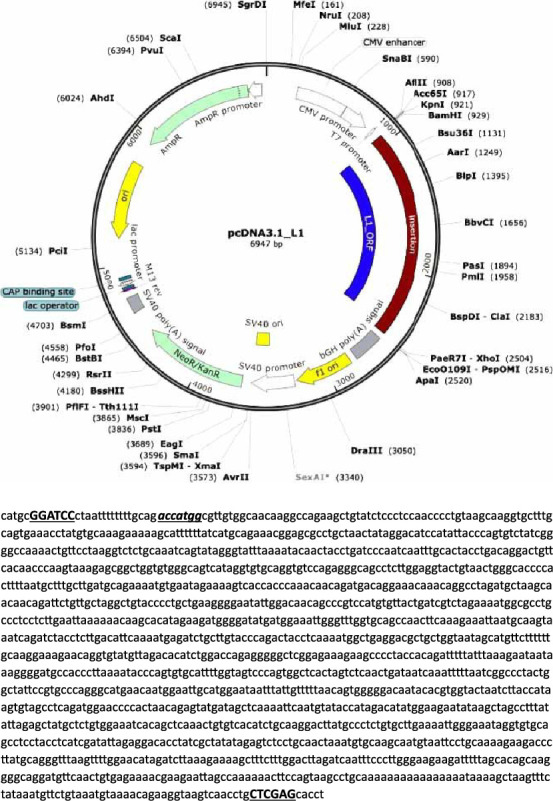
(a) Schematic of the pcDNA3.1_L1 plasmid, full-length, bovine codon–optimized BPV-1 major capsid protein gene cloned into expression vector pcDNA3.1 with significant vector features indicated (b) full-length L1 gene sequence flanked with cloning primers. The underlined letters indicate the *Bam*HI and *Xho*I restriction sites in forward and reverse primers, respectively. Forward primer was optimized to adding Kozak consensus sequence (duple underlined sequence) by the inserted (ACC) sequence.

Ten amplifying PCR products were purified using a DNA cleanup kit (BioRad, USA), and the purified L1 gene was cloned into a pcDNA3.1 vector (purchased from Gene script company, USA) using T4 DNA ligase (Invitrogen, USA) for the ligation reaction (resulting plasmid named pcDNA3.1-L1) (Figure 1). The vectors were transformed by the heat shock method into *E. coli* BL31 (DE3) competent bacterial cells, provided by Dr. Amjed Alsultan (College of Veterinary Medicine/University of Al-Qadisiyah/Iraq), according to Ref. [30]. Positive clones were selected from LB agar plates supplemented with 100 μg/mL kanamycin. Touch colony PCR was performed to verify the presence of inserts. Subsequently, pcDNA3.1-L1 vectors were extracted from 18–16-h grown broths using Mini Prep Plasmid Extraction Kit (Geneaid, Taiwan) according to the manufacturer's instructions. Vectors were digested with *Bam*HI and *Xho*I to confirm the presence of the L1 insert.

### 2.4. Sequences Acquisition and Phylogenetic Analysis

The positive plasmids (pcDNA3.1-L1) were submitted for bidirectional sequencing using the same primers employed for amplification, through the Sanger sequencing method (Macro gen, Seoul, Korea). The Mega XI program (Version 11.0.13) [31] was used to analyze the complete L1 gene sequences including multiple sequence alignment, predictions of putative ORF, molecular weight, and translating to amino acids. Nucleotide similarity searches were conducted using BLAST analysis (https://blast.ncbi.nlm.nih.gov). Genetic distances were estimated using the maximum composite likelihood method [32]. Phylogenetic reconstruction was performed using the poststrip method, within Mega XI software. The confidence values of internal branch nodes were assessed with 1000 bootstrap replicates. Notably, this analysis included 20 complete L1 gene sequences related to BPV genotypes related to PV genera that were obtained from GenBank. Phylogenetic trees were constructed from the alignment of L1 sequences using the Neighbor-joining method. Lastly, the complete 10 L1 sequences obtained from BPV Iraqi strain were deposited in GenBank, where they can be accessed under Accession Nos. PP082031–PP082040, named IrHY1–IrHY10 isolates, respectively.

### 2.5. In Silico Analysis of L1 Protein

The nucleotide of L1 gene sequences was translated to amino acid sequences by utilizing the ExPASy Translate Tool (https://web.expasy.org/translate/). The protein characteristics and the physicochemical properties such as the number of amino acids, molecular weight, isoelectric points, and percentage of strongly basic, acidic, hydrophobic, and polar amino acids, solubility, estimated half-time, instability index, and antigenicity were determined by the ExPASy tool (https://web.expasy.org/protparam) [33] tool. To predict antigenicity default parameters were applied to the server, with a threshold of 0.5 used to identify the antigenic protein. Allergenicity was evaluated to the proposed epitope for vaccine development; the web-based AllerCatPro (Version 2.0) server was utilized (https://www.ddg-pharmfac.net/AllerTOP/data.html) [34, 35]. Protein solubility was assessed with a Protein-Sol online server (https://protein-sol.manchester.ac.uk/) [36].

### 2.6. Secondary and Tertiary Structures *Prediction of L1 Protein*

Secondary structure prediction of the L1 protein was implemented by I-TASSER online server-based algorithms to generate high-quality model predicting the 3D structure and function of protein based on their amino acid sequences (https://zhanglab.ccmb.med.umich.edu/I-TASSER/) [37]. I-TASSER predicted a secondary structure from the RCSB Protein Data Bank (RCSB PDB) based on the *Z*-score. The model hit with the highest *Z*-score was selected and downloaded from the RCSB PDB server under (ID: 3iyj) [38]. Following that, the ChimeraX server program (https://www.rbvi.ucsf.edu/chimerax) was utilized to manipulation L1 protein single-nucleotide polymorphism (SNPs), used comparative modeling with 3iyj PDB file of the L1 protein as the model for construction and refinement of the 3D structure of the L1 protein. The SNP dropping was done by protein sequence editing and the substitutions of the targeting amino acid. Epitope visualization and protein modifications were done based on the guide of program setting and instructions. The quality of L1 crystal structure was verified using the Ramachandran plot and Klash setting within the ChimeraX server. The backbone conformation of protein structures was assessed by analyzing *ϕ* (Phi) and *ψ* (Psi) dihedral angles for each residue with the Ramachandran plot.

### 2.7. B Cell Epitope Identification

We predict the B- and T-cell epitopes depending on Ref. [39]. B-cell epitope prediction is performed to identify the potential L1 protein regions capable of interacting with B lymphocytes and inducing an immune response. Two types of epitopes, linear and discontinuous B-cell epitopes, were predicted in both the ABCpred servers (https://www.imtech.res.in/raghava/abcpred/) [40]. The predicted epitopes were ranked based on their score obtained by the recurrent neural network with 0.5 threshold value. The higher score of the peptide indicated a higher probability of being an epitope. And the BepiPred server (https://tools.immuneepitope.org/bcell/) [41, 42] was used for prediction. Epitopes predicted by both tools were selected as potential B-cell epitopes. For the determination of conformational epitopes, the L1 protein sequence was proffered to both the CBTope server (https://www.imtech.res.in/raghava/cbtope/) [34] and the BepiPred 2.0 server [43]. Common epitopes predicted by both programs were considered. To further assess the antigenicity of the predicted B-cell epitopes, we utilized the VaxiJen 2.0 server (https://www.ddg-pharmfac.net/vaxijen/VaxiJen/VaxiJen.html) [44]. Furthermore, the predicted B-cell epitopes were checked for toxicity, antigenicity, and allergenicity using the ToxinPred, VaxiJen v 2.0, and the AllerTOPv.2.0 [34] servers, respectively.

### 2.8. T Cell Epitope Identification

Cytotoxic T-lymphocyte (CTL) epitopes were predicted to assess the potential of the L1 peptide binding to the Major Histocompatibility Complex (MHC) Class I molecules. The NetMHCpan-4.1 server (https://services.healthtech.dtu.dk/services/NetMHCpan-4.1/) was conducted with default settings and utilized the binding affinity of peptides to cow MHC alleles. For predicting MHC Class II T-cell epitopes, we utilized the IEDB server (https://tools.immuneepitope.org/mhcii/) The IEDB recommended approach employed the consensus method [43], which combines multiple prediction tools including NN-align [45, 46] and SMM-align [47] and uses NetMHCIIpan if no corresponding predictor is available for the molecule [48]. This consensus approach considers a combination of any three out of the four methods, with Sturniolo being the final choice. The expected predictive performances are based on large-scale evaluations of MHC Class II binding predictions, involving studies with over 10,000 binding affinities [49], over 40,000 binding affinities [43], and a comparison of pan-specific methods [50]. To assess the antigenicity of the predicted T-cell epitopes, we employed the Kolaskar and Tongaonkar antigenicity method [51], available within the IEDB analysis resource available at https://tools.immuneepitope.org/bcell/. The standard threshold value of 1.030 was used.

### 2.9. Epitope Conservancy Analysis

The epitope conservation among different strains was assessed using the IEDB epitope conservancy analysis tool, accessible at https://tools.immuneepitope.org/tools/conservancy/iedb_input [52].

## 3. Results

### 3.1. Clinical and Molecular Findings

**Figure 2:**
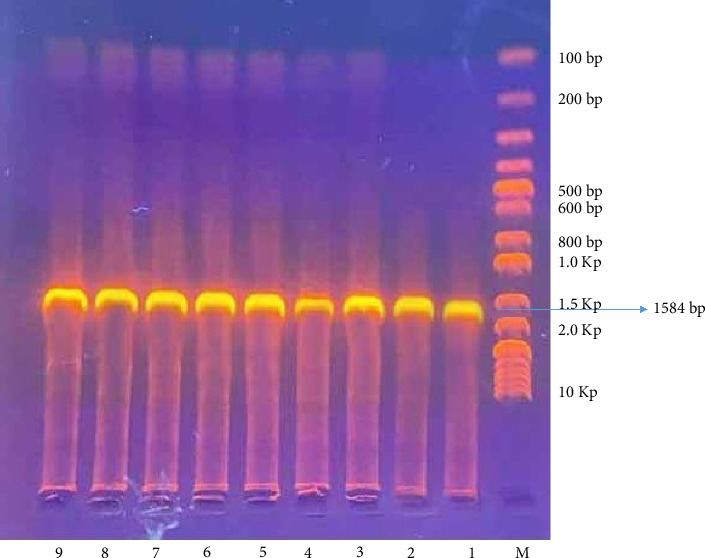
Bovine papillomaviruses–specific primers were used to amplify the whole-length L1 gene (1584 bp). The amplicons were visualized on 1% ethidium bromide impregnated 1% agarose.

A total of 50 skin wart samples were collected from 50 cows in the middle Euphrates region of Iraq, specifically from the provinces of Babylon, Wasit, and Al-Qadisiyah. The choice of cattle breeds was not considered and was contingent upon the availability of available cases. All cows displayed normal temperature, respiration, and appetite. The majority of wart lesions appeared in the abdomen, udder, neck, and face. They measured approximately 1–5 cm in diameter and had gray, hyperkeratotic epidermis. According to the PCR, the results showed that 42 (84%) samples tested positive for BPV; when specific primers were used and the band size was 1584 bp, shown in Figure 2, the remaining eight samples (16%) tested negative.

### 3.2. Genotype Identification, Mutations Determination, and Phylogenetic Analysis

The next step involved cloning and sequencing the L1 gene from 10 positive samples. Sequence analysis revealed that the L1 gene of the BPV identified in the current study matched Delta PV-4/BPV-1 strain and has a distance value of 1%–15% with Delta PV 4 strains globally, and high distance values were observed between our strains and other PV genera (data demonstrated in Supporting table 1). Interestingly, five SNPs have been discovered when compared with close reference strain (ID: X02346.1) in all sequences, which included three synonymous mutations at Nucleotide Positions 183, 285, and 292. And two nonsynonymous mutations resulting in amino acid substitutions (SER31/ASN and Ala55/ASP) as shown in Table 1.

**Figure 3:**
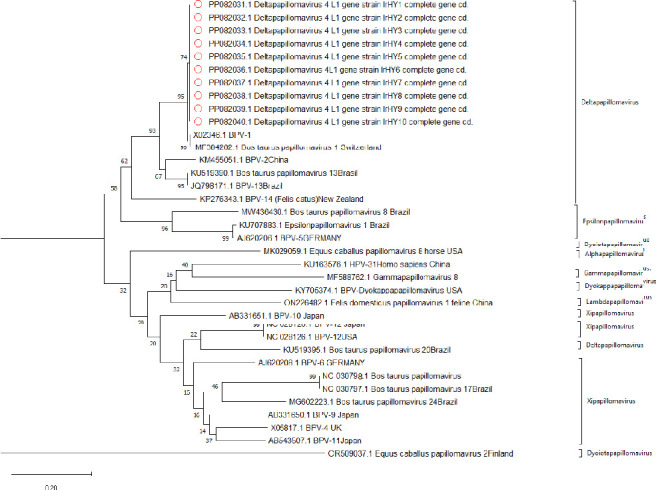
Phylogenetic tree of the Iraqi BPV-1 variants based on the L1 gene. Phylogenetic study was constructed based on 10 whole-length L1 gene sequences (indicted with light cycle) alignment with 20 sequences from different papillomavirus genera, which were constructed by the maximum likelihood method and the Kimura 2-parameter model by MEGA 11. Bootstrap proportions were calculated with 1000 replicates.

Phylogenetic analysis was conducted to determine the evolutionary links between the current BPV-1 strain and other world strains. All 10 sequence samples aligned with 20 strains of various PVs, which had been downloaded from the GenBank, and 12 primary clusters emerged with node robustness percentages equal to or exceeding 70%. The first cluster primarily consisted of BPV-1 types and included our 10 identified genotypes. Intriguingly, these genotypes were exclusively represented by strains from the USA, Switzerland, Brazil, and China (Figure 3).

### 3.3. Amino Acid Sequence Analysis, Antigenicity, and Allergenicity of BPV-L1 Protein

Evaluation of the amino acid sequence was the next target. First, the results of all 10 samples of L1 protein showed 100% homogeneity between them, where the PP082031.1 BPV L1 sequence has been used as the reprehensive for the current sequences. Second, the physiochemical properties of the L1 protein were assessed employing the ExPASy ProtParam tool and Compute pI/Mw-(https://web.expasy.org/compute_pi/). Also, the molecular weight was 55,622.26 Da and the length was 495 amino acid residues. The computed isoelectric point (pI) value was above 7 (8.57), which indicates the alkaline nature of the final product and confirmed a positive charge at neutral pH. Third, the half-life of the BPV-1 protein was evaluated to be 30 h in mammalian reticulocytes (in vitro), more than 20 h in yeast (in vivo), and more than 10 h in *Escherichia coli* (in vivo). According to the instability index (39.66/stable), the vaccine was classified as a stable protein. The aliphatic index was (77.82). The high aliphatic index indicates that the designed vaccine is stable in different temperature ranges. The estimated grand average hydropathy (GRAVY) value is −0.471, and the negative GRAVY value shows that the protein vaccine is hydrophilic protein and interacts well with water molecules (Table 2).

Finally, the analysis of antigenicity and allergenicity was conducted using the VaxiJen 2.0 server (https://www.ddg-pharmfac.net/vaxijen/VaxiJen/VaxiJen.html). The antigenicity value was 0.562 at 0.5 threshold, which indicates that the L1 protein is antigenic, and it was a nonallergen. All these parameters were comparable to L1 protein sequences of reference strains (MF384282 and X02346), as shown in Table 2.

### 3.4. Prediction of Secondary, Tertiary, and Refinement of the L1 Protein

**Figure 4:**
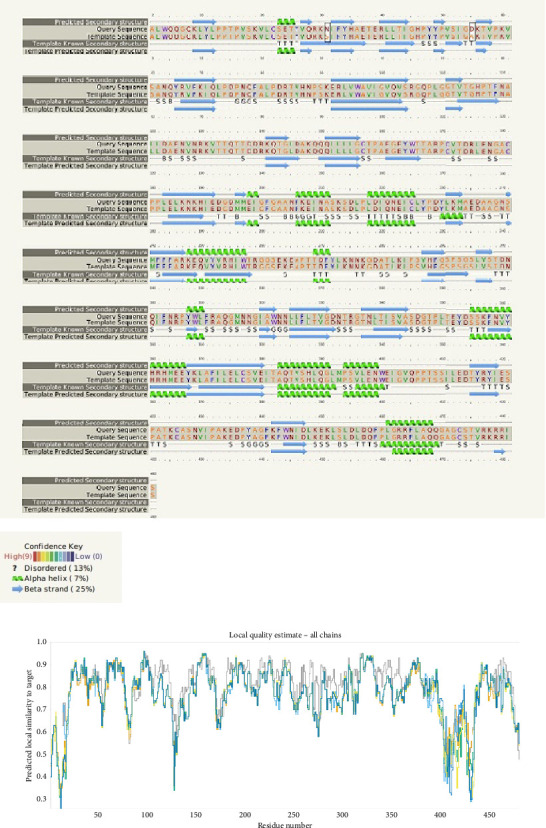
Secondary structure of the BPV-1 L1 protein. Alpha helix (green), beta sheet, and coil with its percentage perdition. Small boxes in 31 and 55 amino acid sequence indicate mutations. Each mutation located at coils.

**Figure 5:**
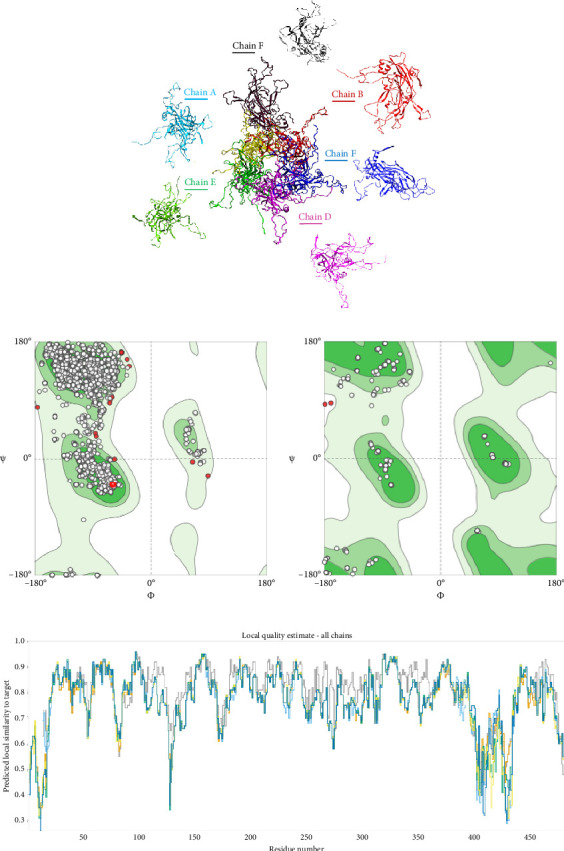
The cartoon structure of BPV Type 1 L1 protein. (a) In central, the tertiary structure of homogeneous hexameric protein, composed of six chains A, B, C, D, E, and F surrounded with each chain alone. (b) Ramachandran plot showed all residues within right regions. (c) Verification of 3D interpretation of L1 protein.

**Figure 6:**
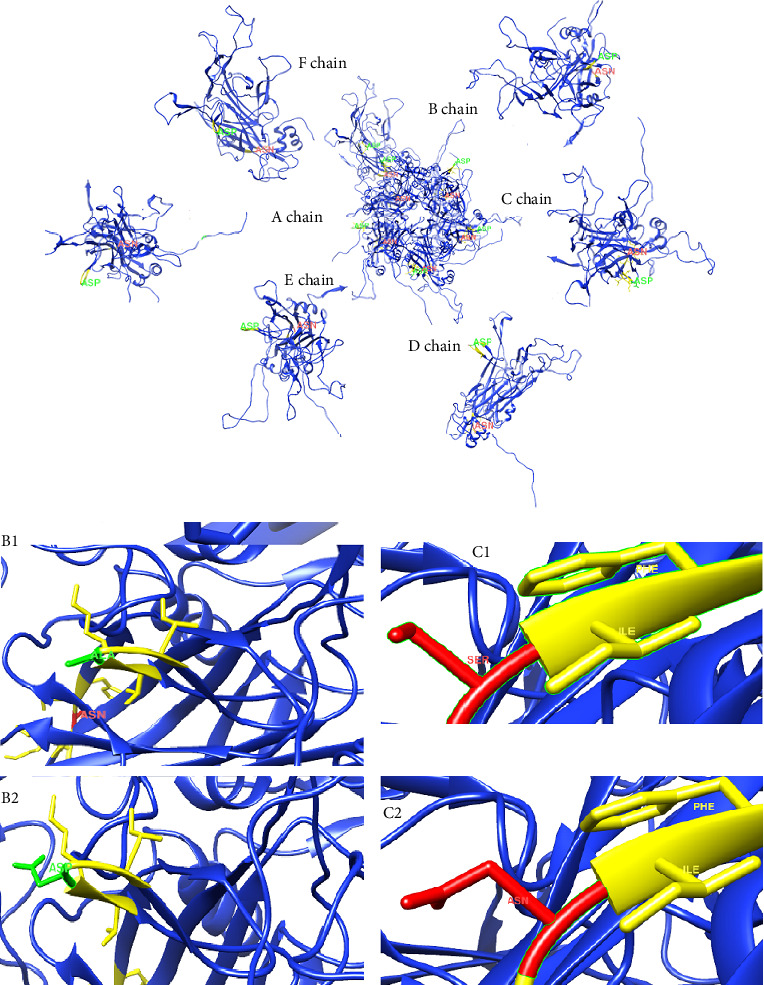
3D carton structures of the L1 protein of BPV-1 Iraqi strain: (A) Hexameric structure of the L1 protein in center surrounding with it chains representing the residues of mutations in 31 and 55 amino acid residues. (B1) ALA amino acids. Mutation residue in control compared with B2 is ASP amino acid in 55 residues (green). (C1) SER amino acid mutation in 31 residue compared with ASN amino acid in reference strain (red).

**Figure 7:**
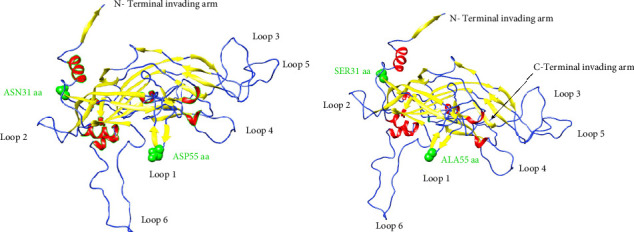
3D structure prediction of BPV-1 L1. (a) Structure of BPV-1 L1 of our study demonstrates the sites of mutations (green color) and (b) control structure.

To predict the protein structure model of the BPV-1 L1 Iraqi strain, I-Tasser was employed. The BPV-1 protein sequence was used as the input with default threshold values. The model with the highest *Z*-score was selected. The secondary structure of our L1 protein included 7% alpha helix, 25% beta strand as shown in Figure 4. The tertiary structure prediction resulted in the generation of distinct structural models' protein orthologs. The highest *C*-score model was selected as the best, which is used for the next step. PDB file (accession no. 3IYJ) BPV Type 1 outer capsid have been used as model. The SWISS-MODEL tool was used for the modeling and refinement of the subjected crystal structure homology. Furthermore, the L1 protein is distinctively knobby, shows a hexameric structure, and consists of six homology chains (A, B, C, D, E, and F), each monomer having six antigenic loops (Loop1–Loop6). Potential neutralizing epitopes are usually found to be conformational sites that are located on these loops as it can be seen in Figure 5. The selected homology models underwent a refinement process in which the UCSF Chimera tool was utilized. This 3D alignment allowed the clear visualization of the amino acid differences within the BPV-1 protein from Iraqi strain with regard to control reference strain. The two mutations, (SER31/ASN and ALA55/ASP) that were identified, both resulted in amino acid substitutions. The first mutation SER31/ASN, was located out loop region, whereas ALA55/ASP, was situated within Loop1. However, analysis of the protein's 3D structure revealed no significant conformational changes attributable to these mutations (Figure 6). This refined homology model was then validated via a structure validation based on the Ramachandran plot of Chimera tool. Importantly, the ALA55/ASP mutation located within the region (Loop1) that may be associated with immunological activity (Figure 7).

### 3.5. Predicted B-Cell Epitopes of L1 Proteins of BPV-1

**Figure 8:**
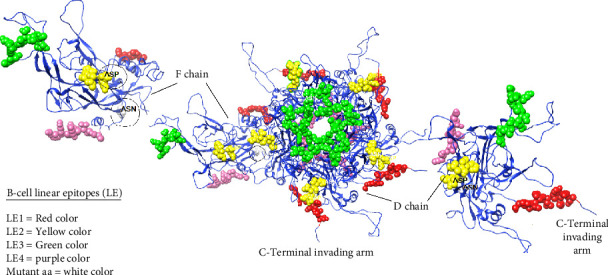
B-cell liner epitopes in the cartoon of BPV Type 1 L1 protein; each epitope is highlighted specific color and labeled with a specific name in all chains of protein, two (F and D) chains are selected to clearly visualize the epitope sites.

Both B-cell epitopes, discontinuous (linear) and continuous (conformational) types, were predicted. First, continuous B-cell epitopes can be predicated by employing the ABCpred and IEDB analysis resource server tools using the Kolaskar and Tongaonkar antigenicity method. Out of 16 predicted linear B-cell epitopes, only four linear epitopes (LE1–LE4) within the L1 protein were selected based on high antigenic scores, nonallergenic, nontoxicity, and positive immunogenicity scores (Table 3). Furthermore, we predicted discontinuous B-cell epitopes (BDE1-BDE5), The conservancy discontinuous B-cell epitopes were assessed using the IEDB conservancy analysis tool with the results presented in Table 4. The conservation rates were 100% for all B-cell epitopes, ensuring their preservation across all BPV-1 protein orthologs. However, we applied the linear B-cell epitopes on the basis of the protein antigen's 3D structure employed SWISS MODEL, which makes it a more reliable method for B-cell epitope prediction visualization as shown in Figure 8. The data analysis showed that ALA55/ASP is located at the region of LE2 located (50–56) residues.

### 3.6. Prediction of Conformational B-Cell Epitopes in the 3D Structure of *L1 Protein*

Conformational B-cell epitopes on the L1 protein were identified by the ElliPro server (accessible via the link https://tools.iedb.org/ellipro/) (51). The server predicts the discontinuous antibody epitopes on the basis of the protein antigen's 3D structure, which makes it a more reliable method for B-cell epitope prediction. However, the PDB file of the protein structure was used as the input data to the ElliPro server. The predicted epitope score refers to the protrusion index (PI) value averaged over epitope residues. The discontinuous epitopes are determined based on the PI values and clustered based on the distance R between the residue's centers of mass. A total of 10 B-cell discontinuous epitopes were predicted (CE1–CE10). The representations of the predicted discontinuous residues on the L1 protein are shown in Supporting table 2. The predicted residues were arranged according to the number of residues, and their score could range from 0.587 to 0.984.

### 3.7. Predicted T-Cell Epitopes of *L1 Protein*

The prediction of MHC Class I T-cell epitopes was performed using the NetMHCpan—4.1 server. The default parameters were kept, and the primary protein sequence was provided as the input. Interestingly, 9–14-mer epitopes were predicted, each with an associated binding affinity score (nM), shown in Table 5. Moreover, all these eight peptides corresponded to the BoLA-HD61 and BoLA-1:00901 haplotypes, presented the highest binding affinity scores, which were < 100 nM, making them a promiscuous MHCI T-cell epitope. For the prediction of epitopes binding to MHC Class II alleles, the NetMHCIIpan—4.1 server was utilized and a total of 9-15-mers epitopes were predicted each with an associate, which binds to MHC Class II allele BoLA-DRB3 ∗ 003:02:01, exhibits a notably high binding affinity score (23–25 nM), making it a potentially good target for subsequent investigations. The conservancy of the predicted T-cell epitopes, of both MHC Class II, was assessed using the IEDB conservancy analysis tool, with the results presented in Table 6. The conservation rate was 100% for all T-cell epitopes, ensuring their preservation across all BPV-1 protein orthologs.

## 4. Discussion

PV capsid proteins L1 usually synthesize late in the infection cycle, often together with L2 protein having crucial roles in viral infectivity and immunogenicity [53, 54]. This study conducted a molecular and computational analysis of the full-length L1 gene of BPV isolates from the middle region of Iraq for the first time. The identified strains were classified as Delta PVs/BPV Type 1, since they are in a close genetic relationship with global BPV-1 strains. In detail, current stains exhibited over 90% sequence identity with global strain [55]. Delta PV 4 is the most prevalent type worldwide [56]. Similarly, the prevalence of BPV-1 was reported previously in Iraqi that reached 86.42% in Iraqi cattle [57]. In addition, previous research conducted in middle and southern provinces of Iraq reported that the majority of cases were classified as BPV-1 type [17, 58].

Understanding the dominant BPV type is essential not only for identifying potential genetic and antigenic alterations that might impact vaccine efficacy but also for tracking the temporal evolution of BPV in Iraq strains. Moreover, this knowledge enables researchers and public health authorities to tailor their responses to the specific efforts posed by this variant. It also offers invaluable data for monitoring the viruses spread within the region and potentially beyond.

In the comparative analysis of L1 gene sequences, with eligible global different PV genera, pairwise sequence identity revealed some of genetic distances. Many publishers have potentially illuminated genetic diversity between PV genera [1]. This finding has significant implications for local and global cattle welfare, emphasizing the importance of understanding the prevalent and characteristics of local viral strains. Such knowledge can profoundly impact disease management and vaccine development [59]. Moving forward, we optimized and cloned a BPV-1 L1 gene (extract from Iraqi strain) into *E. coli* BL31 and the insertion driven by the CMV promoter (pcDNA3.1-L1). We anticipated that vector would be able to enter host cells efficiently by using isotonic solution [60]. Many reports demonstrated that the L1 protein can self-assemble into a structure that closely mimics the natural surface of PV by conserving a native immunogenic property [61]. Therefore, it is the most targeted protein as the basis for highly successful recombinant vaccines against cancer-causing PVs [62].

Notably, the phylogenetic analysis revealed that the 10 sequences in this study formed a distinct cluster closely related to delta PV genera. Five SNPs that can lead to two specific amino acid residue mutation within the L1 protein may play a major role in defining antigenic properties, host specificity, and modulation of the host's immune response [63]. These genetic changes in the virus genome highlight the virus's capacity for genetic evolution.

The 3D modeling of the L1 protein was implemented to assess the impact and sites of both nonsynonymous mutations on the structure of the L1 protein and on vaccine properties. Interestingly, two remarkable mutations found at specific amino acid sites within the L1 protein in comparison with reference strain, one mutation was located within surface loops (Loop1), and it has been assessed to clash and hydrophilicity. The top surface of the L1 capsomer is composed of loop structures. Most of the neutralized antibodies are known to target one or more of these surface loops [61]. These surface loops are extremely variable (poorly conserved) even among closely related viral types. This may be under selective pressure of neutralizing antibodies raised by previous infections [53, 64]. Previous studies with HPVs revealed that amino acid changes within immunodominant epitopes located on surface loops have major biologically importance [65].

Most recently, experimental vaccines have been developed based on genetically modified viruses encoding full-length viral antigens, which induce both humoral and cellular immune responses [25, 27]. Indeed, this advancement in vaccine development is in sync with the development of bioinformatics tools, which, when employed in vaccine studies, can potentially save time, efforts, and resources [28]. In this study, mammalian expressing vector encoding the BPV major capsid protein L1 gene was constructed as the promising target for DNA vaccine design, aiming to elicit an anti-BPV humeral and cellular immune response. Many reports referring to the fact that PV major capsid protein L1 can assemble into capsids in vitro, and the VLP formation of the BPV-1 of the wild-type L1 gene has been seen in vitro cells with an electron microscope [66]. Other reports constructed BPV–VLPs as a platform to present HIV-1 neutralizing epitopes by inserting some HIV epitopes in a certain site of BPV L1 to express the chimeric VLPs presenting the HIV natural epitopes [67]. Many experiments succeed in providing strong humoral and cellular immune responses against human and animal PVs through employed DNA vaccines within animal models [68], and vaccine vectors based on the BPV1 E5 and L2 genes were well expressed in vitro mammalian cells [25].

Vaccines play an important role in safeguarding certain human populations from BPV infection. A PV modified virus vaccine is one of the globally recommended strategies to protecting the young against high risky HPV types of infections in the developed countries [69]. The structural protein L1 represents the primary target for the body's immune defenses by containing specific amino acid sequences known as epitopes. Epitopes on viral particles interact with cell receptors [64]. Since the BPV-1 L1 protein is highly conserved [62], the conservation of epitopes is crucial for covering different viral strains to give a broader protection effect long-lasting immunity response. The realization of this objective was achieved by prioritizing epitopes exhibiting high sequence conservation, a characteristic quantitatively assessed using the IEDB Epitope Conservancy Analysis tool to determine inter-species sequence similarity [42, 70].

It is a perfect target for a prophylactic subunit vaccine. In silico predictions for B-cell epitope prediction were imposed for the L1 amino acid sequences, and it was found that the C-terminal region plays a vital role in generating appropriate immunological responses. These results were closely agreed with the results of Ref. [71]. Surprisingly, eight potential B-cell epitopes within the L1 protein based on their antigenic scores have been identified. These epitopes, which demonstrated the best binding to MHC Class I alleles, were predicted using the NetCTL server. Additionally, we selected the top 11 potential epitopes that bound to MHC Class II alleles using the IEDB server.

Additionally, the study has highlighted the conservation of T-cell epitopes across various BPV-1 strains in Iraq when compared to reference strains. This finding suggests that these preserved T-cell epitopes have significant potential for the development of robust and broadly effective BPV-1 vaccines. Conducting multiepitope vaccine formulations may lead to enhanced immune responses capable of providing protection against a wide array of BPV-1 variants. However, it is imperative to conduct experimental studies to validate these results and assess the practical efficacy of vaccines formulated with these conserved T-cell epitopes.

There is no study reporting the whole LI gene and its mutations for Iraqi strain. Therefore, the current study was considered the first report on the characterization of BPV L1 mutations in Iraqi cattle. Moreover, the genetic diversity of the BPV-1 L1 gene sampled from Iraq was recorded for the first time. Also, our study addresses the prediction of potential immunogenicity of mutant version and how can it be used to stimulate B and T immune cells.

## 5. Conclusion

The current investigation has provided crucial insights into BPV-1–type diversity in the middle provinces of Iraq, and these predominant viruses have been identified and registered at NCBI for the first time. More importantly, these findings have significant implications for bovine health management and control. The amino acid substitutions in the L1 protein have highlighted. Additionally, the conserved T- and B-cell epitopes that which can detect the Iraqi BPV-1 type, were established in the current investigation. Finally, the results presented here construct and evaluate the ability of the L1 viral protein in vivo for transcription and translation. However, current project is the initial phase of creating a DNA-based vaccination for preventative and treatment purposes against BPV-related illnesses.

## Figures and Tables

**Table 1:** The nucleotide sequence alignment and amino acid characterization of the L1 gene indicated five novel mutations, included three nonsynonymous SNP and two synonymous with locations represented with two amino acid changes in the major antigenicity regions compared to other available reference strain (*R*^∗^).

Strains (GenBank accession number)	Nucleotide mutation positions					Amino acid positions	
	Nonsynonymous		Synonymous				
	92	164	183	285	294	31	55
X02346.1 BPV 4, L1 (R)⁣^∗^	G	C	C	G	C	SER	Ala
PP082031.1, BPV 4, L1 gene strain IrHY1	A	A	T	T	T	ASN	ASP
PP082032.1, BPV 4, L1 gene strain IrHY2	A	A	T	T	T	ASN	ASP
PP082033.1, BPV 4, L1 gene strain IrHY3	A	A	T	T	T	ASN	ASP
PP082034.1, BPV 4, L1 gene strain IrHY4	A	A	T	T	T	ASN	ASP
PP082035.1, BPV 4, L1 gene strain IrHY5	A	A	T	T	T	ASN	ASP
PP082036.1, BPV 4, L1 gene strain IrHY6	A	A	T	T	T	ASN	ASP
PP082037.1, BPV 4, L1 gene strain IrHY7	A	A	T	T	T	ASN	ASP
PP082038.1, BPV 4, L1 gene strain IrHY8	A	A	T	T	T	ASN	ASP
PP082039.1, BPV 4, L1 gene strain IrHY9	A	A	T	T	T	ASN	ASP
PP082040.1, BPV 4, L1 gene strain IrHY10	A	A	T	T	T	ASN	ASP

*Note:* The amino acid characterization of L1 indicated novel mutations, some in the region of highest antigenicity compared to other available reference strain (⁣^∗^). Molecular, phylogenetic, and immunoinformatics analyses of L1 gene of Iraqi BPV demonstrated that this antigen and their predicted immunodominant epitopes of B- and T-cells appear to be highly conserved between Iraq isolates and compared to Global and reference strains, suggesting that the minimal intraspecific modifications will not affect the potential cross-protective capacity of humoral and cell-mediated immune responses against multiple BPV worldwide strains.

**Table 2:** BPV-L1 proteins' physiochemical properties.

Protein	Molecular weight	Theoretical pI	Half-life	Instability index (II)	Aliphatic index	Number of amino acids:	Grand average of hydropathy	Antigenicity prediction	Allergenicity prediction (Y/N)	Solubility value
PP082031 BPV L1 protein Iraq strain	55,622.26	8.57	30 h (mammalian reticulocytes, in vitro). > 20 h (yeast, in vivo). > 10 h (*E. coli*, in vivo).	39.66/Stable	77.82	495	−0.471	0.5625	N	0.310
MF384282 BPV L1 protein reference strain	55,551.22	8.68	30 h (mammalian reticulocytes, in vitro). > 20 h (yeast, in vivo). > 10 h (*E. coli*, in vivo).	39.38/Stable	78.02	495	−0.454	0.5599	N	0.310
X02,346 BPV L1 protein reference strain	55,551.22	8.68	30 h (mammalian reticulocytes, in vitro). > 20 h (yeast, in vivo). > 10 h (*E. coli*, in vivo).	39.38/Stable	78.02	495	−0.454	0.5599	N	0.310

**Table 3:** B-cell linear epitopes of the L1 protein predicted using IEDB analysis (threshold: 0.6) and ABCpred (threshold: 0.5) epitope conservancy results.

No.	Name	Start	End	Epitope sequences	Length	Antigenicity	Epitope conservancy (conserved sequence/total)
1.	LE1	7	19	GQKLYLPPTPVS	12	0.7576	100.00% (7/7)
2.	LE2	50	56	PVSIGDK	7	2.9239	100.00% (7/7)
3.	LE3	127	140	NVNRKVTTQTTDD	13	0.5013	100.00% (7/7)
4.	LE4	472	482	GAGCSTVRKRRIS	13	1.2688	100.00% (7/7)

*Note:* The predicted B-cell epitopes are ranked according to their score obtained by the trained recurrent neural network. Higher score of the peptide means the higher probability to be an epitope. All the peptides shown here are above the threshold value chosen.

**Table 4:** B-cell discontinuous epitopes of the L1 protein predicted using BepiPred 2.0 (threshold: 0.6) and CBTope and epitope conservancy result.

Number	Name	Start	End	Epitope sequences	Length	Antigenicity	Epitope conservancy (conserved sequence/total) (%)
1	CE1	11	19	YLPPTPVSK	9	1.1553	100.00
2	CE2	92	100	ERLVWAVIG	9	0.9849	100.00
3	CE3	271	279	FYLKNNKGD	9	0.6345	100.00
5	CE4	472	482	FYLNNKGDATL	11	0.8974	100.00

*Note:* BPV-1 L1 (GenBank Accession No. M74849) was used as the standard reference sequence. Parenthetical notations denote the amino acid changes identified in variant proteins relative to this reference.

**Table 5:** Eight potential T-cell epitopes of the BPV-1 L1 protein binding to MHC Class I alleles were selected based on the basis of their binding score rank predicted by the NetMHCIIpan—4.1 server and epitope conservancy results.

N.	Name	Position	Allele/Haplotype	Peptide sequence (12-mer)	Score	Binding affinity (nM)	Binding affinity rank (%)	Epitope conservancy (conserved sequences/total) (%)
1	TCE1 (II)	27	BoLA-1:00901	VQRKNIFYH	0.927341	215.6	0.02	100.00
2	TCE2 (II)	101	BoLA-HD6	VQVSRGQPL	0.922289	12.30	0.02	100.00
3	TCE3 (II)	280	BoLA-1:00901	ATLKIPSVH	0.875846	99.11	0.06	100.00
4	TCE4 (II)	109	BoLA-HD6	VQVSRGQPL	0.851417	250.63	0.03	100.00
5	TCE5 (II)	147	BoLA-D18.4	AKQQQILLL	0.838614	1333.00	0.01	100.00
6	TCE6 (II)	356	BoLA-1:00902	NQFNYVPSNIGG	0.629306	320.61	0.23	100.00
7	TCE6 (II)	12	BoLA-HD61	ALWQQGQKLYL	0.755908	313.77	0.14	100.00
8	TCE7 (II)	154	BoLA-HD61	KQTGLDAKQQQILL	0.714813	197.11	0.11	100.00

*Note:* Reference sequence BPV-1 L1 (GenBank Accession No. M74849) was utilized for comparative analysis. Amino acid substitutions identified in proteins deduced from variant sequences, relative to the reference, are denoted parenthetically.

**Table 6:** Eleven potential T-cell epitopes of the BPV-1 L1 protein binding to MHC Class II alleles were selected based on the basis of their binding score rank predicted by the NetMHCIIpan—4.1 server and epitope conservancy results.

Allele	Score	Start	End	Length	Core sequence	Epitope sequence	Ic50	Percental rank
BoLA-DRB3 ∗ 002:02	23	1	15	15	IDLKEKLSL	FKFWNIDLKEKLSLD	1448.62	30
BoLA-DRB3 ∗ 005:05	3	3	17	15	YYPVSIGDK	TIGHPYYPVSIGDKT	4476.61	73
BoLA-DRB3 ∗ 004:01	3	5	19	15	YYPVSIGDK	GHPYYPVSIGDKTVP	4757.01	84
BoLA-DRB3 ∗ 003:02:01	25	1	15	15	KTSSKPAKK	ISQKTSSKPAKKKKK	547.06	28
BoLA-DRB3 ∗ 005:03	13	2	16	15	FFFARKEQV	MFFFARKEQVYVRHI	214.23	4.90
BoLA-DRB3 ∗ 003:02:01	24	3	17	15	FLAQQGAGC	RRFLAQQGAGCSTVR	453.28	24
BoLA-DRB3 ∗ 001:01	24	6	20	15	AGCSTVRKR	LAQQGAGCSTVRKRR	1362.97	26
BoLA-DRB3 ∗ 005:05	19	2	16	15	YKLAFILEL	HRHMEEYKLAFILEL	433.16	6.40
BoLA-DRB3 ∗ 004:01	23	2	16	15	IDLKEKLSL	KFWNIDLKEKLSLDL	402.04	26
BoLA-DRB3 ∗ 002:02	1	5	19	15	KLYLPPTPV	QQGQKLYLPPTPVSK	3473.13	57
BoLA-DRB3 ∗ 005:03	1	4	18	15	KLYLPPTPV	WQQGQKLYLPPTPVS	2733.08	69

## Data Availability

The data used to support the findings of this study are available from the corresponding author upon reasonable request.

## Supporting Information

Supporting Information 1 Supporting Table 1: Genetic distances of nucleotides identity rates of full amino acid of 10 sequences of the current study and 20 sequences from different genera of PVs. The numbers on the right and left represent, respectively, the genetic distance and the amino acid identity rates (%) observed between the sequences. All information regarding the sampling country and year associated with the sequence analyzed in this table is presented in the phylogenetic tree shown in Figure 3.

Supporting Information 2 Supporting Table 2: Discontinuous B-cell epitopes of the BPV-1 L1 protein predicted by the ElliPro server based on the protein antigen's 3D structure. The reference protein 3D structure considered in this analysis is predicted from the genetic variant BPV-1 L1 protein.
