# Supplementary material for: Molecular Analysis and Bioinformatics Assessment of Full-Length L1 Gene of Bovine Papillomavirus Type-1 as a Potential DNA Vaccine Study
Source: Vet Med Int. 2025 Apr 10;2025:6785087. doi: 10.1155/vmi/6785087 (PMC12006685; doi:10.1155/vmi/6785087)
Supplement: Supporting Information 1 — Supporting Table 1: Genetic distances of nucleotides identity rates of full amino acid of 10 sequences of the current study and 20 sequences from different genera of PVs. The numbers on the right and left represent, respectively, the genetic distance and the amino acid identity rates (%) observed between the sequences. All information regarding the sampling country and year associated with the sequence analyzed in this table is presented in the phylogenetic tree shown in Figure 3. [file 6785087.f1.docx]

Supplementary table 1

| Strains | PP082031.1 | PP082032.1 | PP082033.1 | PP082034.1 | PP082035.1 | PP082036.1 | PP082037.1 | PP082038.1 | *PP082039.1* | PP082040.1 |
| --- | --- | --- | --- | --- | --- | --- | --- | --- | --- | --- |
| PP082031.1_Deltapapillomavirus_4_L1_gene_strain_IrHY1 |  | 0.000 | 0.000 | 0.000 | 0.000 | 0.000 | 0.000 | 0.000 | 0.000 | 0.000 |
| PP082032.1_Deltapapillomavirus_4_L1_gene_strain_IrHY2 | 0.000 |  | 0.000 | 0.000 | 0.000 | 0.000 | 0.000 | 0.000 | 0.000 | 0.000 |
| PP082033.1_Deltapapillomavirus_4_L1_gene_strain_IrHY3 | 0.000 | 0.000 |  | 0.000 | 0.000 | 0.000 | 0.000 | 0.000 | 0.000 | 0.000 |
| PP082034.1_Deltapapillomavirus_4_L1_gene_strain_IrHY4 | 0.000 | 0.000 | 0.000 |  | 0.000 | 0.000 | 0.000 | 0.000 | 0.000 | 0.000 |
| PP082035.1_Deltapapillomavirus_4_L1_gene_strain_IrHY5 | 0.000 | 0.000 | 0.000 | 0.000 |  | 0.000 | 0.000 | 0.000 | 0.000 | 0.000 |
| PP082036.1_Deltapapillomavirus_4L1_gene_strain_IrHY6 | 0.000 | 0.000 | 0.000 | 0.000 | 0.000 |  | 0.000 | 0.000 | 0.000 | 0.000 |
| PP082037.1_Deltapapillomavirus_4_L1_gene_strain_IrHY7 | 0.000 | 0.000 | 0.000 | 0.000 | 0.000 | 0.000 |  | 0.000 | 0.000 | 0.000 |
| PP082038.1_Deltapapillomavirus_4_L1_gene_strain_IrHY8 | 0.000 | 0.000 | 0.000 | 0.000 | 0.000 | 0.000 | 0.000 |  | 0.000 | 0.000 |
| PP082039.1_Deltapapillomavirus_4_L1_gene_strain_IrHY9. | 0.000 | 0.000 | 0.000 | 0.000 | 0.000 | 0.000 | 0.000 | 0.000 |  | 0.000 |
| PP082040.1_Deltapapillomavirus_4_L1_gene_strain_IrHY10 | 0.000 | 0.000 | 0.000 | 0.000 | 0.000 | 0.000 | 0.000 | 0.000 | 0.000 |  |
| X02346.1_BPV-1 | 0.010 | 0.010 | 0.010 | 0.010 | 0.010 | 0.010 | 0.010 | 0.010 | 0.010 | 0.010 |
| KU163576.1_HPV-31Homo_sapiens_China | 0.725 | 0.725 | 0.725 | 0.725 | 0.725 | 0.725 | 0.725 | 0.725 | 0.725 | 0.725 |
| KU519395.1_Bos_taurus_papillomavirus_20Brazil | 0.712 | 0.712 | 0.712 | 0.712 | 0.712 | 0.712 | 0.712 | 0.712 | 0.712 | 0.712 |
| KU519390.1_Bos_taurus_papillomavirus_13Brasil | 0.143 | 0.143 | 0.143 | 0.143 | 0.143 | 0.143 | 0.143 | 0.143 | 0.143 | 0.143 |
| KU707883.1_Epsilonpapillomavirus_1_Brazil | 0.461 | 0.461 | 0.461 | 0.461 | 0.461 | 0.461 | 0.461 | 0.461 | 0.461 | 0.461 |
| MF384282.1_Bos_taurus_papillomavirus_1_Switzerland | 0.015 | 0.015 | 0.015 | 0.015 | 0.015 | 0.015 | 0.015 | 0.015 | 0.015 | 0.015 |
| MW436430.1_Bos_taurus_papillomavirus_8_Brazil | 0.455 | 0.455 | 0.455 | 0.455 | 0.455 | 0.455 | 0.455 | 0.455 | 0.455 | 0.455 |
| NC_028126.1_BPV-12_Japan | 0.648 | 0.648 | 0.648 | 0.648 | 0.648 | 0.648 | 0.648 | 0.648 | 0.648 | 0.648 |
| NC_030797.1_Bos_taurus_papillomavirus_17_Brazil | 0.796 | 0.796 | 0.796 | 0.796 | 0.796 | 0.796 | 0.796 | 0.796 | 0.796 | 0.796 |
| ON226482.1_Felis_domesticus_papillomavirus_1_feline_China | 0.685 | 0.685 | 0.685 | 0.685 | 0.685 | 0.685 | 0.685 | 0.685 | 0.685 | 0.685 |
| KM455051.1_BPV-2China | 0.167 | 0.167 | 0.167 | 0.167 | 0.167 | 0.167 | 0.167 | 0.167 | 0.167 | 0.167 |
| X05817.1_BPV-4_UK | 0.652 | 0.652 | 0.652 | 0.652 | 0.652 | 0.652 | 0.652 | 0.652 | 0.652 | 0.652 |
| MG602223.1_Bos_taurus_papillomavirus_24Brazil | 0.723 | 0.723 | 0.723 | 0.723 | 0.723 | 0.723 | 0.723 | 0.723 | 0.723 | 0.723 |
| AB543507.1_BPV-11Japan | 0.621 | 0.621 | 0.621 | 0.621 | 0.621 | 0.621 | 0.621 | 0.621 | 0.621 | 0.621 |
| KY705374.1_BPV-Dyokappapapillomavirus_USA | 0.708 | 0.708 | 0.708 | 0.708 | 0.708 | 0.708 | 0.708 | 0.708 | 0.708 | 0.708 |
| AB331650.1_BPV-9_Japan | 0.649 | 0.649 | 0.649 | 0.649 | 0.649 | 0.649 | 0.649 | 0.649 | 0.649 | 0.649 |
| AB331651.1_BPV-10_Japan | 0.501 | 0.501 | 0.501 | 0.501 | 0.501 | 0.501 | 0.501 | 0.501 | 0.501 | 0.501 |
| AJ620208.1_BPV-6_GERMANY | 0.570 | 0.570 | 0.570 | 0.570 | 0.570 | 0.570 | 0.570 | 0.570 | 0.570 | 0.570 |
| NC_028126.1_BPV-12USA | 0.648 | 0.648 | 0.648 | 0.648 | 0.648 | 0.648 | 0.648 | 0.648 | 0.648 | 0.648 |
| NC_030797.1_Bos_taurus_papillomavirus_17Brazil | 0.796 | 0.796 | 0.796 | 0.796 | 0.796 | 0.796 | 0.796 | 0.796 | 0.796 | 0.796 |
| AJ620206.1_BPV-5GERMANY | 0.455 | 0.455 | 0.455 | 0.455 | 0.455 | 0.455 | 0.455 | 0.455 | 0.455 | 0.455 |
| JQ798171.1_BPV-13Brazil | 0.143 | 0.143 | 0.143 | 0.143 | 0.143 | 0.143 | 0.143 | 0.143 | 0.143 | 0.143 |
| KP276343.1_BPV-14_(Felis_catus)New_Zealand | 0.315 | 0.315 | 0.315 | 0.315 | 0.315 | 0.315 | 0.315 | 0.315 | 0.315 | 0.315 |
| MF588762.1_Gammapapillomavirus_8 | 0.813 | 0.813 | 0.813 | 0.813 | 0.813 | 0.813 | 0.813 | 0.813 | 0.813 | 0.813 |
| OR509037.1_Equus_caballus_papillomavirus_2Finland | 1.271 | 1.271 | 1.271 | 1.271 | 1.271 | 1.271 | 1.271 | 1.271 | 1.271 | 1.271 |
| MK029059.1_Equus_caballus_papillomavirus_8_horse_USA | 0.606 | 0.606 | 0.606 | 0.606 | 0.606 | 0.606 | 0.606 | 0.606 | 0.606 | 0.606 |
