# Supplementary material for: Molecular Analysis and Bioinformatics Assessment of Full-Length L1 Gene of Bovine Papillomavirus Type-1 as a Potential DNA Vaccine Study
Source: Vet Med Int. 2025 Apr 10;2025:6785087. doi: 10.1155/vmi/6785087 (PMC12006685; doi:10.1155/vmi/6785087)
Supplement: Supporting Information 2 — Supporting Table 2: Discontinuous B-cell epitopes of the BPV-1 L1 protein predicted by the ElliPro server based on the protein antigen's 3D structure. The reference protein 3D structure considered in this analysis is predicted from the genetic variant BPV-1 L1 protein. [file 6785087.f2.docx]

Supplementery table 2

| Number | Name | Discontinuous B-Cell Epitopes | Number of residues | Score | 3D structure |
| --- | --- | --- | --- | --- | --- |
| 1 | B-DE1 | A:A2, A:L3, A:W4, A:Q5 | 4 | 0.984 | 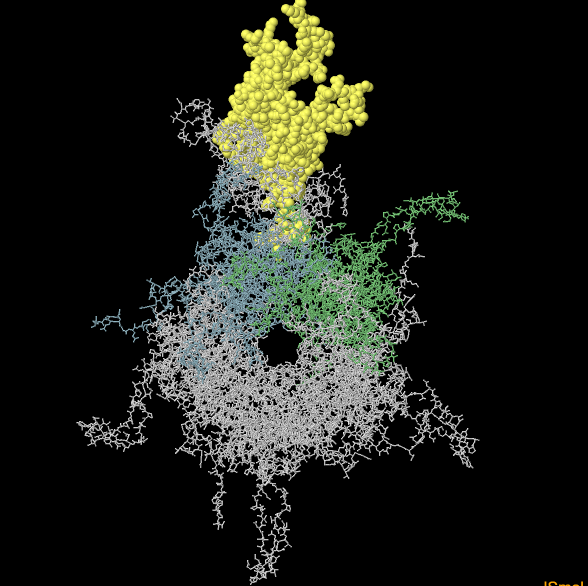 |
| 2 | B-DE2 | E:W4, E:Q5, E:Q6, E:G7, E:Q8 | 5 | 0.947 | 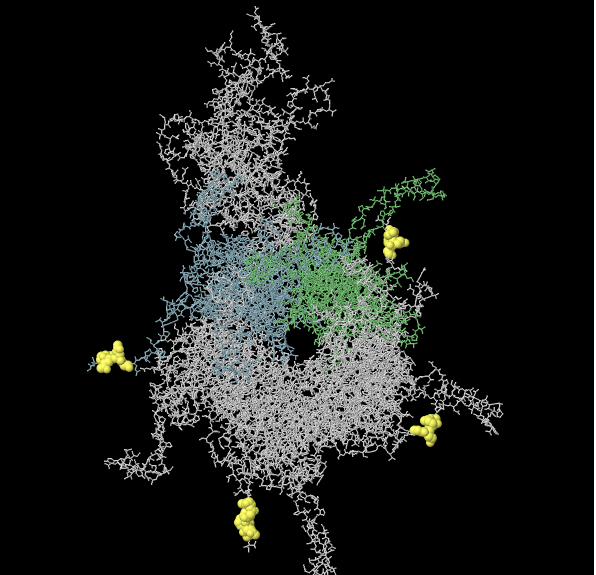 |
| 3 | B-DE3 | A:A80, A:L81, A:P82, A:D83, A:R84, A:T85, A:V86, A:H87, :N88, A:P89, A:S90, A:K91, A:E92, A:L397, A:I402, A:G403, A:V404, A:Q405, A:P406, A:P407, A:T408, A:S409, A:S410, A:I411, A:L412, A:E413, A:D414, A:T415, A:Y416, A:R417, A:Y418, A:I419, A:E420, A:S421, A:P422, A:A423, A:T424, A:K425, A:C426, A:A427, A:S428, A:N429, A:V430, A:I431, A:P432, A:A433, F:P14, F:T15, F:P16, F:V17, F:S18, F:K19, F:V20, F:L21, F:C22, F:S23, F:E24, F:T25, F:Y26, F:V27, F:Q28, F:R29, F:K30, F:S31, F:I32, F:F33, F:Y34, F:H35, F:A36, F:E37, F:T38, F:E39, F:R40, F:L41, F:L42, F:T43, F:I44, F:G45, F:H46, F:P47, F:Y48, F:Y49, F:P50, F:V51, F:S52, F:I53, F:G54, F:A55, F:K56, F:T57, F:V58, F:P59, F:K60, F:V61, F:S62, F:A63, F:N64, F:Q65, F:Y66, F:R67, F:V68, F:F69, F:I71, F:P76, F:N77, F:L81, F:P82, F:D83, F:R84, F:T85, F:V86, F:H87, F:N88, F:P89, F:S90, F:K91, F:E92, F:L94, F:V95, F:W96, F:A97, F:V98, F:I99, F:G100, F:V101, F:Q102, F:V103, F:S104, F:R105, F:G106, F:Q107, F:P108, F:L109, F:G110, F:G111, F:T112, F:V113, F:T114, F:G115, F:H116, F:P117, F:T118, F:F119, F:N120, F:A121, F:L122, F:L123, F:D124, F:A125, F:E126, F:N127, F:V128, F:N129, F:R130, F:K131, F:V132, F:T133, F:T134, F:Q135, F:T136, F:T137, F:D138, F:D139, F:R140, F:K141, F:Q142, F:T143, F:G144, F:L145, F:D146, F:A147, F:K148, F:Q149, F:Q150, F:Q151, F:I152, F:L153, F:L154, F:L155, F:G156, F:C157, F:T158, F:P159, F:A160, F:E161, F:G162, F:E163, F:Y164, F:W165, F:T166, F:T167, F:A168, F:R169, F:P170, F:C171, F:V172, F:T173, F:D174, F:R175, F:L176, F:E177, F:N178, F:G179, F:A180, F:C181, F:P182, F:P183, F:L184, F:E185, F:L186, F:K187, F:N188, F:K189, F:H190, F:I191, F:E192, F:D193, F:G194, F:D195, F:M196, F:M197, F:E198, F:I199, F:G200, F:F201, F:G202, F:A203, F:A204, F:N205, F:F206, F:K207, F:E208, F:I209, F:N210, F:A211, F:S212, F:K213, F:S214, F:D215, F:L216, F:P217, F:L218, F:D219, F:I220, F:Q221, F:N222, F:E223, F:I224, F:C225, F:L226, F:Y227, F:P228, F:D229, F:Y230, F:L231, F:K232, F:M233, F:A234, F:E235, F:D236, F:A237, F:A238, F:G239, F:N240, F:S241, F:M242, F:F243, F:F244, F:F245, F:A246, F:R247, F:K248, F:E249, F:Q250, F:V251, F:Y252, F:V253, F:R254, F:H255, F:I256, F:W257, F:T258, F:R259, F:G260, F:G261, F:S262, F:E263, F:K264, F:E265, F:A266, F:P267, F:T268, F:T269, F:D270, F:F271, F:Y272, F:L273, F:K274, F:N275, F:N276, F:K277, F:G278, F:D279, F:A280, F:T281, F:L282, F:K283, F:I284, F:P285, F:S286, F:V287, F:H288, F:F289, F:G290, F:S291, F:P292, F:S293, F:G294, F:S295, F:L296, F:V297, F:S298, F:T299, F:D300, F:N301, F:Q302, F:I303, F:F304, F:N305, F:R306, F:P307, F:Y308, F:W309, F:L310, F:F311, F:R312, F:A313, F:Q314, F:G315, F:M316, F:N317, F:N318, F:G319, F:I320, F:A321, F:W322, F:L326, F:F327, F:L328, F:T329, F:V330, F:G331, F:D332, F:N333, F:T334, F:R335, F:G336, F:T337, F:N338, F:L339, F:T340, F:I341, F:S342, F:V343, F:A344, F:S345, F:D346, F:G347, F:T348, F:P349, F:L350, F:T351, F:E352, F:Y353, F:D354, F:S355, F:S356, F:K357, F:F358, F:N359, F:V360, F:Y361, F:H362, F:R363, F:H364, F:M365, F:E366, F:E367, F:Y368, F:K369, F:L370, F:A371, F:F372, F:I373, F:L374, F:E375, F:L376, F:C377, F:S378, F:V379, F:E380, F:I381, F:T382, F:A383, F:Q384, F:T385, F:V386, F:S387, F:H388, F:L389, F:Q390, F:G391, F:L392, F:M393, F:P394, F:K442, F:F443, F:W444, F:L452, F:S453, F:L454, F:D455, F:L456, F:D457, F:Q458, F:F459, F:P460, F:L461, F:G462, F:R463, F:R464, F:F465, F:L466, F:A467, F:Q468, F:Q469, F:G470, F:A471, F:G472, F:C473, F:S474, F:T475, F:V476, F:R477, F:K478, F:R479 | 446 | 0.786 | 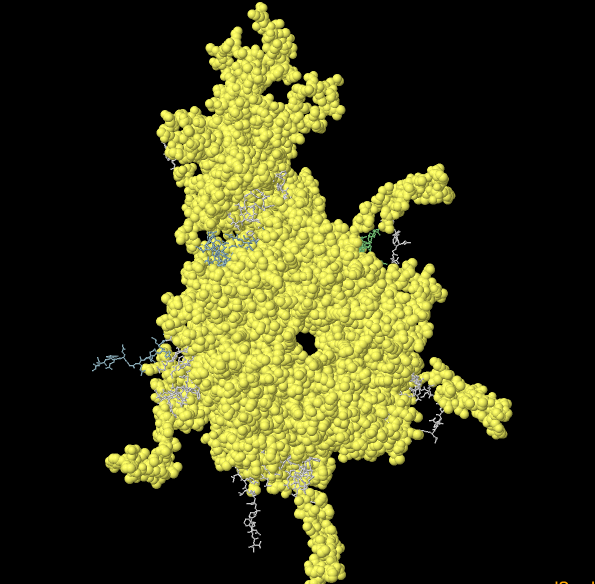 |
| 4 | B-DE4 | B:Y26, B:V27, B:Q28, B:K30, B:S31, B:I32, B:P74, B:D75, B:P76, B:N77, B:Q78, B:F79, B:A80, B:L81, B:P82, B:D83, B:T85, B:V86, B:H87, B:N88, B:P89, B:S90, B:K91, B:E92, B:R93, B:L94, B:L376, B:S378, B:V379, B:E380, B:I381, B:T382, B:A383, B:Q384, B:T385, B:V386, B:S387, B:L397, B:E398, B:N399, B:W400, B:E401, B:I402, B:G403, B:V404, B:Q405, B:P406, B:P407, B:T408, B:S409, B:S410, B:I411, B:L412, B:E413, B:D414, B:T415, B:Y416, B:I419, B:E420, B:S421, B:P422, B:A423, B:T424, B:K425, B:C426, B:A427, B:S428, B:N429, B:V430, B:I431, B:P432, B:A433, B:K434, B:E435, B:D436, B:A439, B:D447, B:K449, B:E450, B:K451, C:Q5, C:Q6, C:G7, C:Q8, C:K9, C:L10, C:Y11 | 87 | 0.742 | 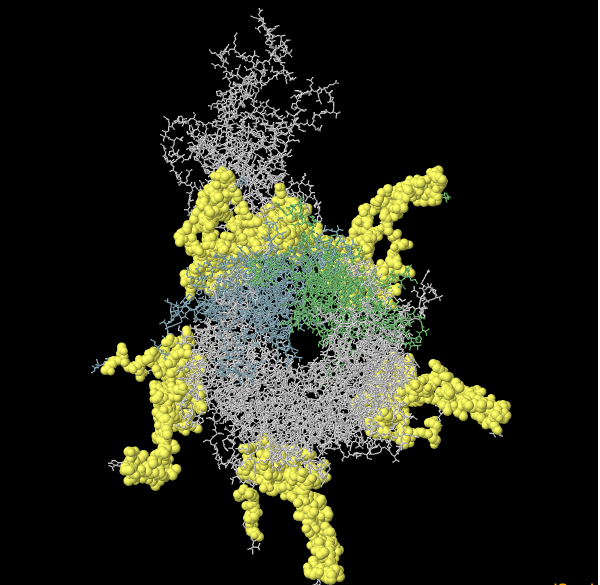 |
| 5 | B-DE5 | A:K9, A:L10, A:Y11, B:Y34, B:L452, B:S453, B:L454, B:D455, B:L456, B:D457, B:Q458, B:F459, B:G470, B:A471, B:G472, B:C473, B:S474, C:L12, C:P13, C:P14, C:T15, C:P16, C:V17, C:S18, C:K19, C:V20, C:L21, C:C22, C:S23, C:E24, C:T25, C:Y26, C:V27, C:Q28, C:R29, C:K30, C:S31, C:I32, C:F33, C:Y34, C:K70, C:I71, C:Q72, C:L73, C:P74, C:D75, C:P76, C:N77, C:Q78, C:F79, C:A80, C:L81, C:P82, C:D83, C:T85, C:V86, C:H87, C:N88, C:P89, C:S90, C:K91, C:E92, C:R93, C:L94, C:V95, C:W96, C:A97, C:C157, C:T158, C:P159, C:A160, C:H190, C:I191, C:E192, C:D193, C:D195, C:D236, C:A237, C:A238, C:G239, C:N240, C:S241, C:F243, C:G315, C:M316, C:N317, C:N318, C:G319, C:I320, C:A321, C:W322, C:N323, C:N324, C:L325, C:L374, C:E375, C:L376, C:C377, C:S378, C:V379, C:E380, C:I381, C:T382, C:A383, C:Q384, C:T385, C:V386, C:S387, C:H388, C:L389, C:Q390, C:G391, C:L392, C:M393, C:P394, C:S395, C:V396, C:L397, C:E398, C:N399, C:W400, C:E401, C:I402, C:G403, C:V404, C:Q405, C:P406, C:P407, C:T408, C:S409, C:S410, C:I411, C:L412, C:E413, C:D414, C:T415, C:Y416, C:I419, C:E420, C:S421, C:P422, C:A423, C:T424, C:K425, C:C426, C:A427, C:S428, C:N429, C:V430, C:I431, C:P432, C:A433, C:K434, C:K442, C:F443, C:N445, C:I446, C:D447, C:L448, C:K449, C:E450, C:K451, C:L452, C:S453, C:L454, C:D455, C:L456, C:D457, C:Q458, C:F459, C:Q469, C:G470, C:A471, C:G472, C:C473, C:S474, D:Q5, D:Q6, D:G7, D:Q8, D:K9, D:L10, D:Y11, D:L12, D:P13, D:P14, D:T15, D:P16, D:V17, D:S18, D:K19, D:V20, D:L21, D:C22, D:S23, D:E24, D:T25, D:Y26, D:V27, D:Q28, D:R29, D:K30, D:S31, D:I32, D:F33, D:Y34, D:H35, D:Q72, D:L73, D:P74, D:D75, D:P76, D:N77, D:Q78, D:F79, D:A80, D:L81, D:P82, D:D83, D:T85, D:V86, D:H87, D:N88, D:P89, D:S90, D:K91, D:E92, D:R93, D:L94, D:V95, D:W96, D:A97, D:C157, D:D236, D:A237, D:A238, D:G315, D:M316, D:N317, D:N318, D:I320, D:A321, D:W322, D:N323, D:N324, D:L325, D:L374, D:E375, D:L376, D:C377, D:S378, D:V379, D:E380, D:I381, D:T382, D:A383, D:Q384, D:T385, D:V386, D:S387, D:H388, D:L389, D:Q390, D:G391, D:L392, D:M393, D:P394, D:S395, D:V396, D:L397, D:E398, D:N399, D:W400, D:I402, D:G403, D:V404, D:Q405, D:P406, D:P407, D:T408, D:S409, D:S410, D:I411, D:L412, D:E413, D:D414, D:T415, D:Y416, D:I419, D:E420, D:S421, D:P422, D:A423, D:T424, D:K425, D:C426, D:A427, D:S428, D:N429, D:V430, D:I431, D:P432, D:A433, D:K434, D:D447, D:L448, D:K449, D:E450, D:K451, D:L452, D:S453, D:L454, D:D455, D:L456, D:D457, D:Q458, D:F459, D:L466, D:Q469, D:G470, D:A471, D:G472, D:C473, D:S474, E:K9, E:L10, E:Y11, E:L12, E:P13, E:P14, E:T15, E:P16, E:V17, E:S18, E:K19, E:V20, E:L21, E:C22, E:S23, E:E24, E:T25, E:Y26, E:V27, E:Q28, E:R29, E:K30, E:S31, E:I32, E:F33, E:Y34, E:K70, E:Q72, E:L73, E:P74, E:D75, E:P76, E:N77, E:Q78, E:F79, E:A80, E:L81, E:P82, E:D83, E:T85, E:V86, E:H87, E:N88, E:P89, E:S90, E:K91, E:E92, E:R93, E:L94, E:V95, E:W96, E:A97, E:C157, E:T158, E:P159, E:A160, E:K189, E:H190, E:E192, E:D193, E:D236, E:A237, E:A238, E:G239, E:N240, E:S241, E:F243, E:G315, E:M316, E:N317, E:N318, E:I320, E:A321, E:W322, E:N323, E:N324, E:L325, E:L374, E:E375, E:L376, E:C377, E:S378, E:V379, E:E380, E:I381, E:T382, E:A383, E:Q384, E:T385, E:V386, E:S387, E:H388, E:L389, E:Q390, E:G391, E:L392, E:M393, E:P394, E:S395, E:V396, E:L397, E:E398, E:N399, E:W400, E:E401, E:I402, E:G403, E:V404, E:Q405, E:P406, E:P407, E:T408, E:S409, E:S410, E:I411, E:L412, E:E413, E:D414, E:T415, E:Y416, E:I419, E:E420, E:S421, E:P422, E:A423, E:T424, E:K425, E:C426, E:A427, E:S428, E:N429, E:V430, E:I431, E:P432, E:A433, E:K434, E:E435, E:D436, E:P437, E:Y438, E:A439, E:G440, E:F441, E:K442, E:F443, E:N445, E:D447, E:L448, E:K449, E:E450, E:K451, E:L452, E:S453, E:L454, E:D455, E:L456, E:Q458 | 481 | 0.74 | 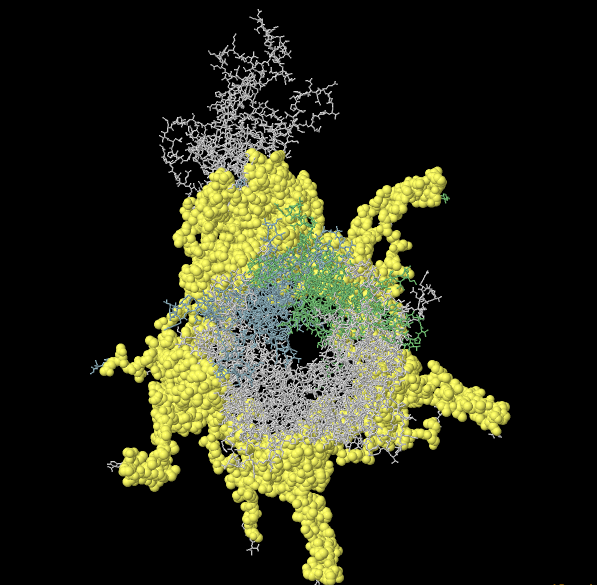 |
| 6 | B-DE6 | C:E435, C:D436, C:P437, C:Y438, C:A439, C:G440, C:F441 | 7 | 0.723 | 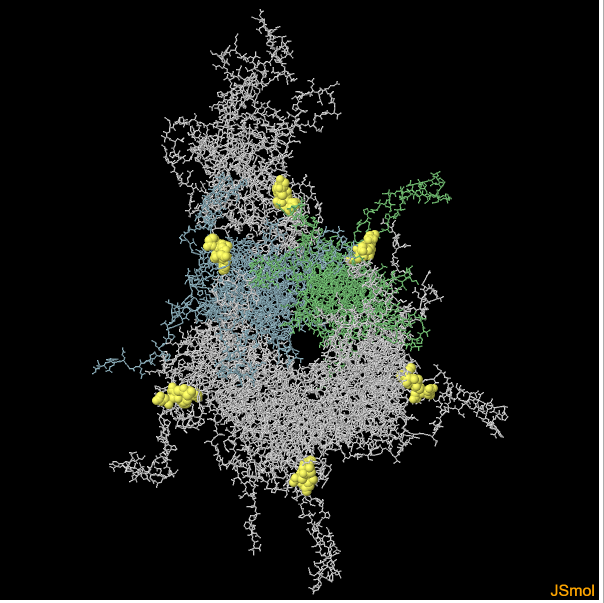 |
| 7 | B-DE7 | A:L12, A:P13, A:P14, A:T15, A:P16, A:V17, A:S18, A:K19, A:V20 | 9 | 0.684 | 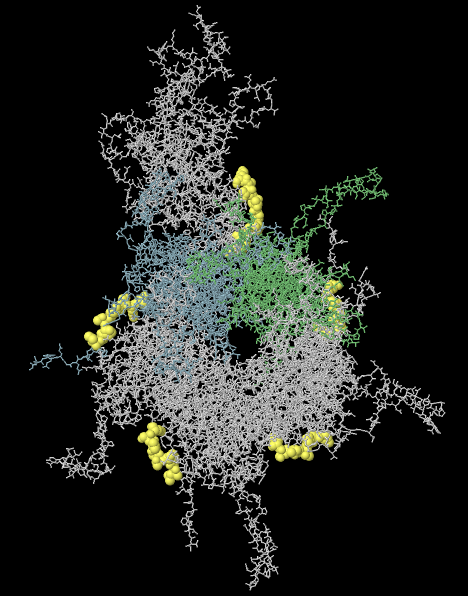 |
| 8 | B-DE8 | B:P50, B:V51, B:S52, B:I53, B:G54, B:A55, B:K56, B:T57 | 8 | 0.619 | 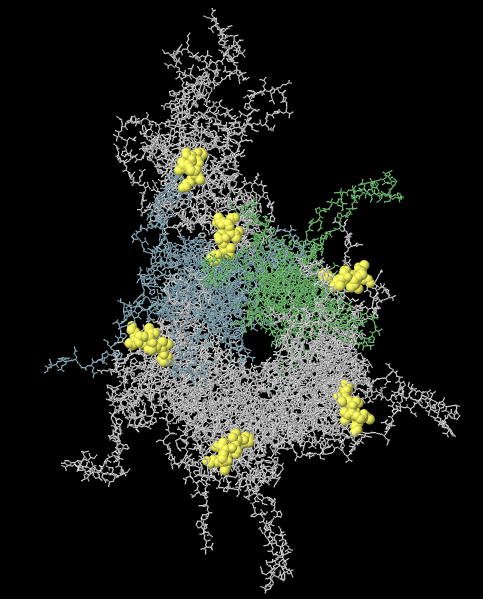 |
| 9 | B-DE9 | D:Y49, D:P50, D:V51, D:S52, D:I53, D:G54, D:A55, D:K56, D:T57, D:V58, D:P59, D:K60 | 12 | 0.60 | 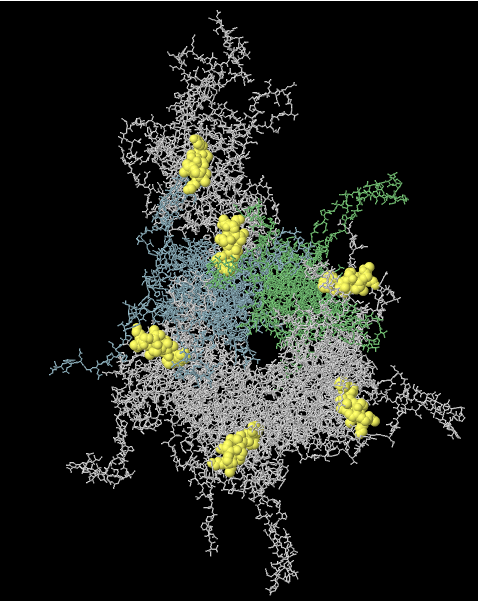 |
| 10 | B-DE10 | C:H46, C:Y48, C:Y49, C:P50, C:V51, C:S52, C:I53, C:G54, C:A55, C:K56, C:T57, C:V58, C:P59, C:K60, C:V61 | 15 | 0.587 | 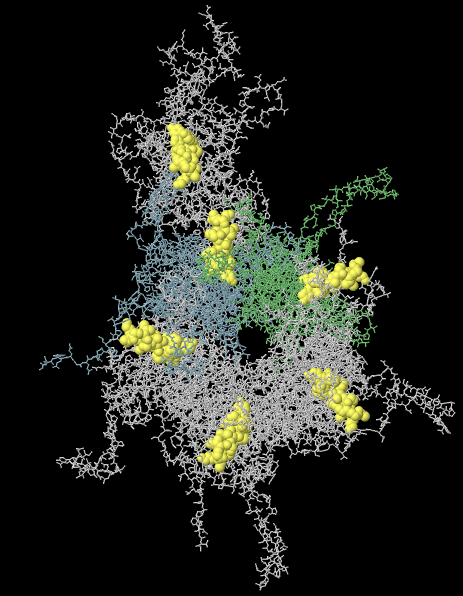 |
